# Optimizing Biomedical Health Efficiency: Unlocking the Full Potential of Life Science Innovation Through System Design

**DOI:** 10.1007/s43441-025-00847-2

**Published:** 2025-07-26

**Authors:** Gigi Hirsch, Sharon E. Phares, Jane Barlow, Murray Aitken, Mark Cziraky, Gregory Daniel, Chester Good, Annie Kennedy

**Affiliations:** 1 Tufts Medical Center, Institute for Clinical Research and Health Policy Studies, Center for Biomedical System Design & NEWDIGS, Tufts Medical Center, Center for Biomedical System Design & NEWDIGS, Boston, USA; 2 Real Endpoints, Florham Park, USA; 3 IQVIA (United States), Durham, USA; 4 Carelon Research, Wilmington, USA; 5 Eli Lilly and Company, Indianapolis, USA; 6 UPMC Health Plan, Pittsburgh, USA; 7 EveryLife Foundation for Rare Diseases, Washington, USA

**Keywords:** Biomedical innovation, System design, Health efficiency, Metrics, Collaborative design, Healthcare system

## Abstract

Major advances in biomedical science have transformed healthcare. However, system barriers to the appropriate, timely, and equitable use of biomedical innovations have led to slow and inconsistent adoption, limiting and delaying our ability to leverage their full potential to improve health. System barriers include inconsistent coverage, imperfect information systems for decision making and real word evidence, policy constraints, system capacity, social influences on health, and infrastructure gaps.

We propose the development of an open access dynamic design “engine” to align biomedical and health system innovation. This engine will include coordinated collaborative design processes, frameworks, and tools, developed with input from all stakeholders, and centered around two critical, interdependent capabilities: (1) system design and (2) impact measurement. These capabilities will build capacity for efficient, model-driven design and implementation planning of sustainable, patient centered system innovations.

The stakes are high for both the clinical promise of transformational products and their budget impact. Our current healthcare system is not ready to maximize benefit from transformational science and emerging biomedical innovations. We need to help the healthcare system catch up with the science.

## Introduction

Over fifty years of major advances in biomedical science including drug therapies, cell and gene therapies, vaccines, diagnostics, and medical technology have transformed healthcare [1–4]. However, system barriers to the timely, appropriate and equitable use of biomedical innovations have often led to slow and inconsistent adoption [5–7], limiting or delaying our ability to leverage their full potential to improve health.

Despite high spending, the U.S. consistently ranks near the bottom among other high-income countries around the world in healthcare outcomes, with lower life expectancy, higher infant and maternal mortality, and higher rates of avoidable and preventable deaths [8, 9]. This is more than just lost opportunity for patients. Failure to leverage the full potential of biomedical advances undermines the credibility of life science innovation and the global leadership of the U.S. in science, technology, and innovation– the cornerstones of the American economy [10]. 

Capturing the full value of biomedical products requires getting the “right treatments to the right patients at the right time” [11] to optimize health outcomes in ways that are operationally practical and economically sustainable for patients, clinicians, provider systems, health systems, pharmacy benefit managers, payers, pharmacies, and life science companies. The U.S. healthcare system is plagued with an estimated $800B of waste and inefficiency [12]. Paying for the potential value of biomedical innovations that we are unable to fully capture fuels the perception that innovation simply drives cost and more waste without additional value to the system [13]. 

Life science companies (a single stakeholder) create the intrinsic value of biomedical products through their innovation research and development process [14]. In contrast, unlocking the full potential value of the products to improve health requires the collective timely impact of the decisions and actions of multiple stakeholders across the healthcare system. The collective impact on value capture can be enhanced or constrained by how the system (e.g., processes, policies, technologies, human capital) is designed, and how these design features affect stakeholder behaviors.

Understanding the current system barriers to value capture is important. For example, drug value assessments that fail to consider these barriers may overestimate the budget impact of new products, leading to coverage policy restrictions designed to manage budget impact, which in turn, exacerbates access challenges that undermine the value impact of the technology [15]. 

As the number of potentially transformational biomedical products entering the market grows, it has become increasingly clear that the system is not ready for the science. Healthcare system innovation and the policies that govern it are lagging behind the pace of scientific innovation in ways that are costly both clinically and economically to all stakeholders in the system. We need a new way forward to fully leverage the potential value of biomedical products to improve health outcomes for patients in ways that work for stakeholders essential in improving people’s health.

We propose the development of a new approach– Biomedical Health Efficiency - which aligns biomedical and health system innovations. This would take a systems thinking approach to design processes, frameworks, and tools, coupled with an impact measurement framework that assesses progress and gaps in efforts to optimize patient outcomes and the value of biomedical innovation.

## The Challenge of Leveraging the Full Value of Biomedical Innovation

Shared value capture involves creating solutions that benefit patients, communities, and other relevant stakeholders. However, the U.S. healthcare system is shaped by dynamic and interdependent relationships between delivery systems, providers, patients, life science companies, payers, policymakers, pharmacy benefit managers, care management providers, and interest group backgrounds– each with their own nuanced definition of value. This complex interplay of stakeholders means that no single organization or stakeholder alone can effectively design more efficient ways to improve health in scalable ways. However, despite the need for cooperation, collaborative success is challenging due to misaligned and often conflicting stakeholder incentives, as well as inertia from the perceived burden of change in an already overburdened system.

System design has an outsized impact on our ability to leverage the full value of biomedical products due to factors such as:

- **Lack of Systematic Data Collection and Data Sharing to Generate the Real-World Evidence (RWE)** needed to reduce clinical uncertainty and allow adaptive decision-making in coverage and reimbursement of innovations. For example, despite FDA approval of lipid-lowering proprotein convertase subtilisin kexin type 9 (PCSK9) inhibitors a decade ago [16] and their use being included in the American College of Cardiology (ACC)/American Heart Association (AHA) cardiovascular treatment guidelines [17], utilization remains low. Payers initially enforced strict prior authorization criteria on the use of these medications, as the clinical effectiveness data on meaningful outcomes (reduction in cardiovascular events) were not felt to be justified by the cost [18]. Even with significantly lowered prices and the eventual relaxation of coverage criteria, the delay in providing clear RWE benefits for PCSK9 inhibitors continues to severely inhibit their use [19, 20].
- **Policy Constraints** such as statutory restrictions on Medicare Part D coverage of anti-obesity medications (AOMs) including GLP-1 agonists that have demonstrated effectiveness in producing substantial reductions in weight. More than 20% of Part D enrollees have a medical diagnosis of obesity and one estimate found that the 10-year cumulative cost offsets from Medicare coverage would exceed $175 billion [21]. Additionally when including social benefit, the value of covering AOM’s would be more be than one trillion dollars. Because of policy constraints however, less than 1% of adults ages 60–69, meeting clinical criteria for treatment for obesity with GLP-1s, are currently receiving treatment [13]. Policy constraints are also based on evidence questions that arose from earlier products as is the case with the two marketed amyloid targeted therapies to treat Alzheimer’s Disease. CMS still utilizes a restrictive Coverage with Evidence (CED) policy, blocking coverage unless patients agree to participate in a registry or randomized control trial [22], despite these therapies receiving traditional approval by the FDA, plus additional publications demonstrating clear evidence that supports coverage [23–26].
- **System Capacity**,** Social Influences on Health**,** and Infrastructure Gaps** such as workforce shortages, geographic differences in access to care, and complex manufacturing requirements create barriers to the optimal use of biomedical innovation. For example, CAR-T cell therapies are clinically effective in treating certain blood cancers with over 80% of patients achieving complete remission [27]. However, despite the approval of 18 genetically modified cell therapies by the FDA for cancer and genetic blood disorders, use has been considerably lower than expected with only 38% of referred patients receiving treatment [28]. This is due in large part to a system constraint - provider capacity to provide these treatments [29]. CAR-T therapies require a workforce of both clinical providers and non-clinical support staff, along with the infrastructure to provide the specialized support needed for patients receiving them [30]. Similarly, patients with rare diseases such as spinal muscular atrophy, particularly those in rural and low-income areas, may not benefit from innovations such as nusinersen due to geographic distance from specialized centers and a shortage of qualified providers [31, 32].

## A New Approach

How do we accelerate and optimize collective impact to capture the value of biomedical products more fully at scale and with greater efficiency? The U.S. healthcare system needs to prioritize the development of system innovations that enhance value using a collective impact approach. This requires multi-stakeholder collaboration around a common agenda, interactive design processes, and shared measurement, applying some key principles of systems engineering. We believe that this is an essential new competency for healthcare systems, requiring first the recognition of the opportunity and then a novel approach to address it.

It is clear that success will require collaboration among stakeholders. At the same time, pilot activities are likely to be designed, governed, and implemented locally. Building upon an understanding of change management in complex systems [33, 34], our proposed approach strives to make it easier for various groups to self-organize around distributed innovation activities that are likely to advance shared success.

We propose the multistakeholder co-creation of a new Biomedical Health System design “engine” to align biomedical and health system innovation. This engine will include coordinated collaborative design processes, frameworks, and tools, developed with input from all stakeholders, and centered around two critical, interdependent capabilities: (1) system design and (2) impact measurement. These capabilities will build capacity for efficient, model-driven design and implementation planning of sustainable, patient centered system innovations.

The engine could build capacity for high impact, rapid-cycle system innovation in two critical and interdependent dimensions: (1) system design and (2) impact measurement. Coupling design and metrics is crucial for ensuring that innovations are both effective and efficient toward our shared goal of collective impact. Integration helps align design with performance goals, enhancing the overall impact and sustainability of any system change being implemented.

Including multiple stakeholders is critical to both the design and management in this approach. First, and foremost, it is essential for ensuring incentive alignment in solution design. In addition, multi stakeholder involvement leverages diverse perspectives, resources, and expertise to address complex health challenges more effectively. It also uncovers “blind spots” in incentive alignment and impact on other parts of healthcare delivery that might not be seen by a single stakeholder group.

## Design of “Precision Access Systems”

We propose focusing design methods and tools on Precision Access Systems (PAS), recognizing that improving access and use of biomedical products requires a systems approach which includes coordinated and targeted advancements across multiple dimensions, such as payment models, evidence generation, point of care implementation, and patient participation.

There are existing methods that could be helpful in system design [35, 36], but they are not routinely used in healthcare innovation, nor are they applied by multi-stakeholder groups. Additionally, these methods would need adaptation to the specific challenges of shared value capture from biomedical products.

Targeting design objectives that are aligned with key tenets of organizational change management [37, 38] will help to catalyze adoption of the system innovations developed. Critical objectives include aligning incentives across stakeholders and making it easier (less friction, fewer delays, fewer resources) for everyone to do the right things for patients.

## Measurement of “Biomedical Health Efficiency”

In coordination with system design, we see the opportunity to address gaps in measurement that are essential for enabling incentive alignment across stakeholders. This new Biomedical Health Efficiency (BHE) metrics framework will be developed with input from all stakeholders and in collaboration with patient community experts. As design efforts identify key leverage points in the system for driving the desired collective impact, the associated metrics will be defined in ways where success requires alignment of stakeholders’ decisions and behaviors, centered around the goal of optimizing patient outcomes in ways that work for everyone.

When combined with PAS design tools, BHE metrics should help to answer questions such as:

- Where do value leaks occur across the patient journey?
- Is one solution or another more effective in capturing shared value?
- How well is the system able to leverage value as determined by other measures (i.e., value assessments)?

BHE could serve as a powerful adjunct to value assessments, offering insights into gaps between value projected vs. impact delivered. Additionally, improving the collection and sharing of real-world usage patterns and evidence would allow value assessments to be refined to reflect performance in current use as well as identify gaps for improvement.

The critical importance of improving data collection and sharing cannot be underestimated. The lack of availability, fragmentation, and low quality of the health data available today leads to the metrics currently being often poorly suited [39]. This can lead to a negative influence on determinations of value and health decision making. We believe that metrics need to be meaningful to all stakeholders and developed with their input. This would avoid some of the current challenges. For instance, total cost of care (TCOC) is often touted as an important measure. However, TCOC is also seen as too hard to measure, plagued by lack of data availability, has a high likelihood of measurement error, lacks standardization, fails to consider the value of health improvements, and lacks consistent association to quality of care [40–43]. By identifying gaps in the current system and then implementing system changes to make fit-for-purpose data collection and tracking easier, new metrics that capture costs as well as health outcomes could be measured more readily, enabling assessments that are currently not feasible.

## Conclusion

While it may be tempting to try to address barriers to capturing the full value of biomedical innovation by “going for low hanging fruit first” in order to “deliver a quick win,” this piecemeal approach to system innovation is notoriously flawed [44, 45]. A key tenet to our approach is that identifying and addressing targeted system inefficiencies cannot be done in scalable and sustainable ways one solution or one stakeholder at a time. One-off point solutions fuel unintended consequences and exacerbate fragmentation and inefficiency. Solutions driven by a single stakeholder may incentivize their gains at the expense of other stakeholders, as value to one party often means risk to another. Thus, solutions require mitigating risk and aligning incentives around shared value to make doing the right things for patients easier for everyone.

The key principles of the BHE framework we propose here are that solution development should be developed by multiple stakeholders, guided and coordinated by a neutral intermediary, with solutions that deliver near-term disease specific value while building enduring capacity. The crucial role of neutral intermediaries in bringing collaborators together has been demonstrated in the success of projects like C-Path [46], which was launched by the FDA, but operates as a safe harbor for stakeholders from the biopharmaceutical industry, academia, regulators, and patients to accelerate the discovery and of new treatments. Other U.S. examples include the Clinical Trials Transformation Initiative (CTTI), a public-private partnership co-founded by the FDA and Duke University to improve clinical trial quality and efficiency, with the Duke Research Institute acting as neutral intermediary [47]. The Milken Institute’s FasterCures began with a U.S. focus to accelerate medical research by aligning interests of stakeholders across sectors and operates as an independent, neutral intermediary with diverse funding sources [48]. In recent years FasterCures has distinguished itself as having a much more global reach. Examples from non-U.S. countries include Quebec’s healthcare sector Knowledge Sharing and Skills Development Initiative [49, 50], Health Innovation Manchester in the United Kingdom [51], and the Innovative Health Initiative (IHI, formerly known as the Innovative Medicines Initiative [52]. The NEWDIGS multistakeholder consortium [53] is currently prototyping BHE concepts in Alzheimer’s Disease and Obesity Management.

Although this paper focused primarily on the U.S., BHE could be applied in any country or region. Since the inputs for health systems vary by country to some extent, particularly in resources available, health policy, and approach to data collection, these should be considered in the context appropriate for a given geographic area. However, the concept remains the same. Achieving BHE results in better outcomes for patients and good stewardship of resources. In a multi-payer environment such as the U.S., BHE and the tools used to test the impact of potential pilot activities on all stakeholders, will allow innovation to be “derisked” to some extent in advance and ensure that potential changes improve access for patients while preserving healthy competition.

The stakes are high for both the clinical promise of transformational products and their budget impact. Finding ways to navigate the current barriers is a survival issue, not only for patients but also for payers, provider systems, clinicians, and life science companies. Just as biomedical products are evolving rapidly– so too is our fundamental understanding of the diseases targeted by emerging products. Decades of research led to the shift of obesity from a behavioral or moral problem to a complex disease with genetic, metabolic, environmental, and behavioral influences [54]. An early diagnosis of Alzheimer’s Disease is no longer to be avoided and stigmatized but is essential for optimizing the clinical value of emerging therapies [55, 56]. New regulatory approved therapeutics can be scientifically exciting, but require coordinated changes in clinical care models, real-world learning systems, health system infrastructures, cost-effectiveness research, and policies to leverage their full clinical value to patients. They also require the progressive reduction of uncertainty through outcomes tracking and RWE production to ensure that the patients who receive the products (alone or in combination) are those who will benefit from them the most.

Simply put, our current healthcare system is not ready to benefit from transformational science and emerging biomedical innovations. In fact, most will say the healthcare system is broken and that it cannot be fixed. We believe that we cannot afford to not fix it.

Efforts to align biomedical and health system innovation in order to capture the full value of biomedical innovations are vitally important and are proliferating. That said, it is simply not possible to get where we need to go one pilot at a time. We are more likely to succeed in timely, scalable, and sustainable ways through strategic collaboration on essential design and measurement tools.

Emerging life science innovations have the potential to fundamentally transform health. We need to help the system catch up with the science.

## Data Availability

No datasets were generated or analysed during the current study.
